# Targeting glycolysis in non-small cell lung cancer: Promises and challenges

**DOI:** 10.3389/fphar.2022.1037341

**Published:** 2022-11-30

**Authors:** Jia-Qi Xu, Yan-Li Fu, Jing Zhang, Kai-Yu Zhang, Jie Ma, Jing-Yi Tang, Zhi-Wei Zhang, Zhong-Yan Zhou

**Affiliations:** ^1^ Longhua Hospital, Shanghai University of Traditional Chinese Medicine, Shanghai, China; ^2^ Department of Oncology, Shenzhen (Fu Tian) Hospital, Guangzhou University of Chinese Medicine, Guangdong, China; ^3^ State Key Laboratory of Pharmaceutical Biotechnology and Department of Pharmacology and Pharmacy, The University of Hong Kong, Hong Kong, Hong Kong SAR, China

**Keywords:** metastasis, tumor microenvironment, aerobic glycolysis, glucose transporter, pyruvate kinase M2, natural product, chemotherapy resistance, biopharmaceutical therapy

## Abstract

Metabolic disturbance, particularly of glucose metabolism, is a hallmark of tumors such as non-small cell lung cancer (NSCLC). Cancer cells tend to reprogram a majority of glucose metabolism reactions into glycolysis, even in oxygen-rich environments. Although glycolysis is not an efficient means of ATP production compared to oxidative phosphorylation, the inhibition of tumor glycolysis directly impedes cell survival and growth. This review focuses on research advances in glycolysis in NSCLC and systematically provides an overview of the key enzymes, biomarkers, non-coding RNAs, and signaling pathways that modulate the glycolysis process and, consequently, tumor growth and metastasis in NSCLC. Current medications, therapeutic approaches, and natural products that affect glycolysis in NSCLC are also summarized. We found that the identification of appropriate targets and biomarkers in glycolysis, specifically for NSCLC treatment, is still a challenge at present. However, LDHB, PDK1, MCT2, GLUT1, and PFKM might be promising targets in the treatment of NSCLC or its specific subtypes, and DPPA4, NQO1, GAPDH/MT-CO1, PGC-1α, OTUB2, ISLR, Barx2, OTUB2, and RFP180 might be prognostic predictors of NSCLC. In addition, natural products may serve as promising therapeutic approaches targeting multiple steps in glycolysis metabolism, since natural products always present multi-target properties. The development of metabolic intervention that targets glycolysis, alone or in combination with current therapy, is a potential therapeutic approach in NSCLC treatment. The aim of this review is to describe research patterns and interests concerning the metabolic treatment of NSCLC.

## Introduction

Today, cancer is the greatest worldwide public health problem after cardiovascular and cerebral vascular disease (Bray et al., 2021). According to the latest epidemiological statistics from 2020, the global incidence of cancer is 19.3 million and mortality is 9.9 million, with lung cancer occupying the second-highest incidence rate (11.4%) and the highest mortality rate (18%) (Sung et al., 2021). Lung cancer has been divided into two types, small cell lung cancer (SCLC) and non-small cell lung cancer (NSCLC), according to pathological diagnosis. Eighty-five percent of lung cancer cases are NSCLC (Gridelli et al., 2015), which includes three subtypes: adenocarcinoma, squamous cell carcinoma, and large cell carcinoma. Although NSCLC generally has better prognosis and slower rate of progression than SCLC, about 50% of patients are diagnosed with local progression or metastasis (Molina et al., 2008; Niu et al., 2016; Rudin et al., 2021). Despite the development of various therapeutic approaches—including classical chemotherapy, targeting therapy, and immunotherapy—the five-year survival rate of metastatic NSCLC in the United States was still under 5% over the past decade (Arbour and Riely, 2019). The discovery of new drugs for NSCLC treatment thus remains an urgent issue.

Emerging evidence indicates that cancer is a metabolic-associated disease. Metabolic disturbances involving glucose, glutamine, and ketone bodies, and particularly energy metabolism supplied by glucose, have been reported as features of tumors progression (Phan et al., 2014; Seyfried et al., 2014; Reinfeld et al., 2021). Targeting metabolic reprogramming (e.g., energy metabolism) is a novel rationale for metabolic drug development in cancers, including in NSCLC (Wu et al., 2015; Park et al., 2020). Glucose is the main substance in intracellular energy metabolism during glycolysis, which produces CO_2_ or lactate under aerobic or anaerobic conditions, respectively, with glycolysis generally referring to anaerobic glycolysis (Lunt and Vander Heiden, 2011). In normal physiology, cells only exhibit glycolysis under oxygen-limited conditions, while cancer cells tend to exhibit glycolysis even under aerobic conditions; this is called the Warburg effect or aerobic glycolysis and distinguishes cancer cells from normal cells (Vander Heiden et al., 2009; Phan et al., 2014; Bose and Le, 2018). Briefly, although aerobic glycolysis is not an efficient means of producing adenosine triphosphate (ATP) compared to oxidative phosphorylation, cancer cells still reprogram the metabolism into glycolysis to meet the high demands of proliferation (Vaupel et al., 2019). Aerobic glycolysis has been considered the fundamental feature of tumor metabolism disturbance and not simply the result of a passive response to mitochondrial damage (DeBerardinis et al., 2008; Wise and Thompson, 2010; Santos and Schulze, 2012). This metabolic reprogramming facilitates tumor survival, which can be viewed as an essential hallmark of cancer (Ward and Thompson, 2012). Glycolysis is critical for energy supply in tumors, as it is the most preferential approach toward energy production (Moreno-Sánchez et al., 2007). Furthermore, glycolysis promotes acidification of the tumor microenvironment (TME), leading to drug resistance (Bose and Le, 2018). Similarly, enhanced metabolism of aerobic glycolysis has been observed in tumors by measurement of intra-operative ^13^C-glucose infusions in NSCLC patients (Hensley et al., 2016). Therefore, targeting the inhibition of glycolytic metabolism could be a potential therapeutic strategy in cancers like NSCLC (Ganapathy-Kanniappan and Geschwind, 2013).

In the current review, we focus on advances in the glycolytic metabolism of NSCLC and provide an overview of key enzymes, biomarkers, and related pathways, as well as the impact of current treatments and candidates in glycolysis.

## Glycolytic metabolism in tumor cells

**FIGURE 1:**
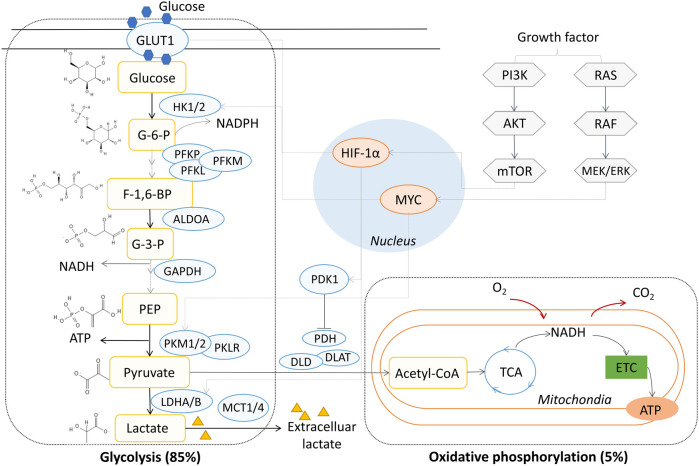
Aerobic glycolysis in NSCLC. GLUT1, glucose transporter 1; HK, hexokinase; PFKL, phosphofructokinase, liver type; PFKM, phosphofructokinase, muscle type; PFKP, phosphofructokinase, platelet type; PKM2, pyruvate kinase M2; PKM1, muscle isoform pyruvate kinase M1; PKLR, red cell/liver pyruvate kinase; LDH, lactate dehydrogenase; MCT1-4, monocarboxylate transporter 1-4; PDK1, pyruvate dehydrogenase kinase 1; PDH, pyruvate dehydrogenase; DLD, dihydrolipoamide dehydrogenase; DLAT, dihydrolipoamide acetyltransferase; ATP, adenosine triphosphate; NADH, nicotinamide adenine dinucleotide; PI3K, phosphoinositide 3-kinase; TCA, tricarboxylic acid; ETC, electron transfer chain; MYC, MYC proto-oncogene; HIF-1α, hypoxia-inducible transcription factor-1 alpha; G-6-P, glucose 6-phosphate; F-1,6-BP, fructose 1,6-phosphate; G-3-P, glyceraldehyde 3-phosphate; PEP, phosphoenolpyruvate.

Glucose is the major source of energy metabolism and biomass synthesis (Lunt and Vander Heiden, 2011). Extracellular glucose crosses the cell membrane with the help of glucose transporter 1 (GLUT1), which also determines the production of glucose 6-phosphate in the first central metabolism pathway mediated by hexokinase 1/2 (HK1/2) (Rajas et al., 2019). Glucose metabolism results in the production of two three-carbon pyruvate molecules from each glucose molecule through a series of enzyme-catalyzed reactions. Pyruvate participates in various physiological metabolic pathways and has several metabolic fates, such as fermentation, cellular respiration, and fatty acid synthesis (Prochownik and Wang, 2021). In normal cells, when oxygen is sufficient, pyruvate is the substrate of acetyl-CoA synthesis, which generates 36 molecules of ATP during the TCA cycle via oxidative phosphorylation in mitochondria. Nicotinamide adenine dinucleotide (NADH) is generated from the TCA cycle, and ATP is produced most efficiently through the phosphorylation of ADP, accompanied with the transformation of NADH to NAD^+^ through the electron transfer chain (ETC) in the mitochondrial inner membrane. This process consumes oxygen and produces CO_2_ from the breakdown of pyruvate. When the oxygen supply is insufficient, pyruvate tends to be metabolized to lactate in anaerobic glycolysis; this process generates lactate and two molecules of ATP, which is independent on the TCA cycle during mitochondrial respiration (Sieow et al., 2018). In tumor cells, aerobic glycolysis occurs and is characterized by 5% oxidative phosphorylation and 85% glycolysis under oxygen-sufficient conditions (Vander Heiden et al., 2009; Fan et al., 2019), and most of the energy supplied is powered by anaerobic glycolysis instead of oxidative phosphorylation (Zheng, 2012). Unlike anaerobic glycolysis in normal cells, aerobic glycolysis is not the passive consequence of hypoxic stress but is rather due to the active metabolic reprogramming of tumor cells (Xu et al., 2020) (Figure 1). Although aerobic glycolysis is not the most efficient means of supplying energy, the proliferation and survival of tumor cells is benefited by glycolysis, which relies on the consumption of intracellular glucose with access to a variety of materials for cell growth, such as nucleotides, amino acids, and lipids (Pavlova and Thompson, 2016; Schiliro and Firestein, 2021). Although glycolysis appears to have weaker energy productivity compared to oxidative phosphorylation, the ATP/ADP and NADH/NAD^+^ ratios usually remain high in tumor cells, indicating sufficient ATP supply in glycolytic tumor metabolism (Martínez-Reyes and Chandel, 2021). The accumulation of lactate facilitates acidification in the TME. The imbalance of nutrient partitioning, as in glutamine metabolism, further enhances the degree of glycolysis, leading to a repeating cycle (Damiani et al., 2017; Reinfeld et al., 2021).

## Key glycolysis enzymes in NSCLC

Glucose metabolism reprograming, as evident in the dependence on glycolysis, is a metabolic hallmark and prognosis parameter of NSCLC, as higher levels of whole-body metabolic tumor volume (MTV) and whole-body total lesion glycolysis (TLG) have been found in NSCLC patients with poor survival rates, based on F-18 fluorodeoxyglucose positron emission tomography (FDG-PET) (Chen et al., 2012; Vanhove et al., 2018). In addition, a high level of TLG was the most accurate risk factor for recurrence in a stage-I postoperative NSCLC patient as compared with the standardized uptake value index (SUV index) and MTV (Melloni et al., 2013). A variety of glycolytic enzymes is aberrantly activated during glycolytic metabolism in NSCLC. We reviewed most of the key enzymes in this study.

### Glucose transporter

Glucose transporter 1 (GLUT1) functions in extracellular glucose uptake and has been proposed as a promising therapeutic target in cancer (Sizemore et al., 2018). In NSCLC, the heterogeneity of GLUT1 is displayed in its subtypes. FDG-PET shows higher MTV and TLG values in GLUT1-positive adenocarcinomas patients as compared to negative cases. Koh et al. found that high expression of GLUT1 presented in 99% of squamous cell carcinoma and 50% of adenocarcinoma patients (Koh et al., 2017a). Human NSCLC samples, patient-derived xenografts, mouse models, and The Cancer Genome Atlas (TCGA) have all consistently showed high expression levels of GLUT1 in squamous cell carcinoma (Goodwin et al., 2017; Smolle et al., 2020). These studies indicate that high expression of GLUT1 is a risk factor for low survival rate in squamous cell carcinoma and adenocarcinoma in NSCLC. In addition, AKT-GLUT1/HKII-targeted microRNA miR-124 overexpression inhibits proliferation, glycolysis, and energy metabolism in A549 cells (Zhao et al., 2017). The Th2-related cytokine IL-33 has been shown to promote patient-derived NSCLC cell growth and metastasis in a nude mice model, with activation of GLUT1 and enhancement of glycolysis (Wang et al., 2016).

### Hexokinase

Hexokinase (HK) plays a vital role in tumor glycolytic metabolism and includes HK1 and HK2 isoforms. It phosphorylates the sixth carbon site of glucose, producing glucose 6-phosphate, and is primary in the pentose phosphate pathway and in glycogen metabolism (Ciscato et al., 2021). The HK1 and HK2 proteins are both highly expressed in *KRAS*-mutant mouse lung tumors, while the activation of HK2 but not HK1 is inevitable for tumor initiation and progression (Patra et al., 2013). In addition, HK2 has been adopted as an essential metabolic marker for the prognostic spectrum of NSCLC, as detected in circulating tumor cells (CTCs) in NSCLC patients (Ma et al., 2020). HK2 is closely associated with survival in NSCLC, as it is involved in the primary step of glucose metabolism. HK2 is required for tumor initiation in the *KRAS*-driven NSCLC mouse model, and the deletion of HK2 prolongs the survival of mice (Patra et al., 2013).

### Rate-limiting enzyme phosphofructokinase

Phosphofructokinase (PFK) is the rate-limiting enzyme converting fructose 6-phosphate to fructose 1,6-bisphosphate. There are three types of phosphofructokinases: muscle type (PFKM), liver type (PFKL), and platelet type (PFKP). In NSCLC, Wang F. et al. (2021) found that PFKP expression was correlated with lymph node metastasis and that high expression of PFKP reduced apoptosis and promoted glycolysis and cell proliferation in H1299 cells. Shen et al. found PFKP to be highly expressed in tissue samples of NSCLC patients and in the PC-9, NCI-H1650, NCI-H520, NCI-H460, H1975, HCC827, and A549 cell lines. The silencing of PFKP inhibited cell proliferation and cell-cycle progress in NCI-H1650 and A549 cell lines (Shen et al., 2020). Additionally, PFKM is prognostic predictor in postoperative NSCLC patients, according to genetic polymorphism research (Lee et al., 2016).

### Pyruvate kinase

Pyruvate is an essential metabolite in glycolysis and has various metabolic fates. The blockage of pyruvate kinase slows the transformation of phosphoenolpyruvate to pyruvate. Pyruvate kinases include the muscle isoforms pyruvate kinase M1 (PKM1) and pyruvate kinase M2 (PKM2), and the liver and red blood cell isoform (PKLR). PKM2, which acts as a nuclear factor and participates in a metabolic loop with GLUT1 (Pan et al., 2019), is highly expressed in NSCLC and is potentially a specific target in treatment of NSCLC, according to data from nine patient-derived cell lines, two established cell lines (H1299 and H358), and nude mice (Suzuki et al., 2019). Hypoxia exacerbates the resistance effects of cisplatin in A549 cells by transmitting exosomal PKM2 to sensitive cells (Wang D. et al., 2021). Pyruvate dehydrogenase (PDH) is one of the key enzyme complexes responsible for the oxidative decarboxylation of pyruvate. Pyruvate dehydrogenase kinase 1 (PDK1), which could inactivate PDH and is an independent risk factor for NSCLC, is highly expressed in tumor tissues of NSCLC patients, and its overexpression promotes the proliferation and metastasis of NSCLC (Liu and Yin, 2017).

### Glyceraldehyde 3-phosphate dehydrogenase

Although glyceraldehyde 3-phosphate dehydrogenase (*GAPDH*) is assumed to be a housekeeping gene, it is a common enzyme with uncommon functions, including glycolysis (Nicholls et al., 2012). GAPDH, an irreversible metabolic switch in glycolysis, catalyzes the conversion of glyceraldehyde 3-phosphate to 1,3-bisphospoglycerate, accompanied by the production of NADH (Liao et al., 2019). *GAPDH* transcription was upregulated in an NSCLC patient cohort and correlated with the glycolysis and gluconeogenesis pathways (Wang et al., 2013). Silencing of GAPDH by RNA interference induced the senescence of A549 cells and enhanced the therapeutic effects of antimetabolite drugs (Phadke et al., 2013). The ratio of GAPDH to mitochondrially encoded cytochrome c oxidase I (GAPDH/MT-CO1) and the ratio of mitochondrial metabolism transcriptional coactivator to peroxisome proliferator-activated receptor-gamma coactivator (PGC)-1 alpha have been considered as biomarkers for the Warburg effect for evaluating relative drug usage benefits in stage-Ⅲ NSCLC patients (Cruz-Bermúdez et al., 2017).

### Lactate dehydrogenase

Lactate dehydrogenase (LDH), which is classified into the two isoforms LDHA and LDHB, is responsible for the mutual transformation between pyruvate and lactate. Intracellular pyruvate is reduced to lactate by LDHA, and then lactate is transported extracellularly, resulting in an acidic TME. On the other hand, LDHB catalyzes the oxidation of lactate to pyruvate (Brisson et al., 2016), which partially promotes the recruitment of intracellular pyruvate. In a meta-analysis study, Deng et al. (2018) found that in lung cancer patients, a high blood LDH concentration was correlated with poor overall survival rate. However, serum LDHB-positive NSCLC patients presented higher recurrence-free survival rates than did LDHB-negative cases, particularly those of the squamous cell carcinoma subtype (Koh et al., 2017b). Deletion of the *LDHB* gene inhibited tumor initiation and progression through mitochondrial DNA damage in a genetically engineered NSCLC mouse model with combined *p53* knockout and *KRAS (G12D)* overexpression (Deng et al., 2022; Stine et al., 2022).

### Monocarboxylate transporter

Monocarboxylate transporters (MCTs) belong to the SLC16A family. MCT1-4 facilitates the membrane-crossing exchange of lactate (Payen et al., 2020). MCT1 and MCT4 are the main functional isoforms of MCTs in cancer and are associated with tumor invasion and metastasis (Sun X. et al., 2020). In NSCLC, overexpressed MCT4 was detected in P29mtB82M human cancer cells (Takenaga et al., 2021), and MCT4-neutralizing antibodies significantly inhibited cell proliferation in glycolysis-preference CL1-5 and Hop62 cells in a concentration-dependent manner (Kuo et al., 2020). Additionally, MCT2 and GLUT1 were reported to be significantly co-overexpressed in adenocarcinomas relative to other NSCLC subtypes (Giatromanolaki et al., 2017).

In summary, key glycolytic enzymes play critical roles in cell proliferation, invasion, and metastasis in NSCLC. Most of the key glycolytic enzymes anomalously expressed in NSCLC could be potential drug development targets. In particular, LDHB may serve as a specific prognostic predictor in the squamous cell carcinomas subtype of NSCLC. The function of LDHB remains ambiguous in other tumor cells, such as triple-negative breast cancer (TNBC) (Naik and Decock, 2020). In addition, targeting both MCT2 and GLUT1 might be promising in the treatment of the adenocarcinoma subtype of NSCLC. Furthermore, PFKM might be a prognostic predictor in postoperative NSCLC patients.

## The regulation and biomarkers of glycolysis in NSCLC

Since glycolysis metabolism reprogramming is common in cancers including NSCLC, the identification of biomarkers and regulators associated with key glycolytic enzymes contributes to the discovery and evaluation of effective therapeutic approaches, particularly biomarkers correlated with the diagnosis and outcome prediction of NSCLC.

### Glycolytic enzyme-regulated biomarkers

#### GFPT2

The glucose uptake-related gene glutamine-fructose-6-phosphate transaminase 2 (*GFPT2*) encodes the rate-limiting enzyme glutamine-fructose-6-phosphate aminotransferase 2 (GFAT2) in the hexosamine biosynthesis pathway. GFPT2 is a GLUT1-independent prognostic predictor in NSCLC patients and functions as a key glucose-uptake mediator. Overexpressed *GFPT2* has been detected in cancer-associated fibroblasts in lung adenocarcinoma and, specifically, regulates metabolic reprogramming in this NSCLC subtype (Zhang et al., 2018).

#### SIX1

Sine oculis homeobox homolog 1 (SIX1) is a transcription factor associated with aerobic glycolysis during tumor growth. SIX1 has been shown to be highly expressed in A549 cells, and SIX1 knockdown in mouse embryos has resulted in the decreasing protein activities of various glycolysis-related enzymes such as HK2, GLUT1, and LDHA (Li et al., 2018a).

#### IL-33

Overexpression of IL-33, which has been suggested to correlate with tumor progression in humans, facilitates tumor growth and metastasis by upregulating GLUT1 and, consequently, enhancing glycolysis in NSCLC nude mice (Wang et al., 2016).

#### DPPA4

Developmental pluripotency-associated 4 (DPPA4) was found to be a poor prognostic factor in NSCLC patients due to the enhancement of glycolytic metabolism *via* increased activity of LDHB, HK2, and GLUT4 (Li L. et al., 2019).

#### NQO1

NADPH quinone oxidoreductase 1 (NQO1) was reported to be a poor prognostic biomarker based on metabolomic analysis in NSCLC A549 cells (Cheng et al., 2018). NQO1 was overexpressed in NSCLC, and the knockdown of the NQO1 gene expression by its specific siRNA inhibited cell proliferation and tumor glycolysis metabolism in A549 cells by downregulating HK2 expression (Cheng et al., 2018).

#### OCT-1

The high expression of octamer transcription factor-1 (OCT-1) is a key feature associated with poor survival rate in NSCLC patients. Glycolysis metabolism was shown to be facilitated by the overexpression of OCT-1 and activation of HK2 in H1299 cells (Li et al., 2021).

#### CD147/basigin

CD147 or basigin (BSG) acts as a molecular chaperone and is responsible for the transport of lactate from the cytoplasm to the plasma membrane, followed by MCT-mediated transport to extracellular matrix. The MCT1/4 expression level and intracellular glycolysis rate were reduced in CD147/BSG-knockout A549, H1975, and H292 cell lines (Granja et al., 2015).

### Transcription factors MYC and HIF-1α and their related molecules

Hypoxia-inducible transcription factor-1 alpha (HIF-1α) is a transcription factor overexpressed in multiple cancer types and plays an important role in lung cancer metabolism by promoting tumor survival (Jun et al., 2017). MYC proto-oncogene (MYC) is a pan-cancer oncogene and is aberrantly amplified in lung cancer (Massó-Vallés et al., 2020). Most of the key glycolytic enzymes are affected by MYC and HIF-1α in tumor glycolysis metabolism (Stine et al., 2015; Soni and Padwad, 2017).

#### TRAF6

Tumor necrosis factor receptor-associated factor 6 (TRAF6) is a biomarker linked with poor prognosis in NSCLC patients (Sun et al., 2019). Downregulation of *TRAF6* gene expression by specific shRNA-mediated RNA interference produced anti-tumor effects in the NSCLC cell lines A549 and NCI-H358 and in A549-transplanted BALB/ca nude mice through the activation of the Akt-HIFα pathway (Feng et al., 2021). In *TRAF6*-knockdown cells, HK2 decreased and deficient HIF-1α was induced, and intracellular glycolysis metabolism was attenuated, accompanied by glucose consumption and lactate production (Feng et al., 2021).

#### KLF5

Knockdown of Krüppel-like factor 5 (*KLF5*) gene expression in NSCLC cell lines H1299 and A549 relieved chemotherapy (cisplatin) resistance mediated by hypoxia, in which HIF-1α was suppressed, *via* inhibition of the phosphoinositide 3-kinase (PI3K)/AKT/mammalian target of rapamycin (mTOR) signaling pathway (Gong et al., 2018).

#### NOX4

NADPH oxidase 4 (*NOX4*) was highly expressed in A549 cells and promoted c-Myc-dependent glycolysis *via* the activation of the reactive oxygen species (ROS)/PI3K/Akt signaling pathway and the pentose phosphate pathway (Zeng et al., 2016).

#### RFP180

Ring finger protein 180 (RFP180) is a potential anti-tumor target, and low expression of RFP 180 indicates poor survival rates in NSCLC patients (Ding et al., 2022). Upregulation of RFP180 impaired glycolysis and proliferation in H292 cells, and similar anti-tumor effects of RFP180 were also shown in the H358 cell-injected xenograft nude mice model (Ding et al., 2022). The fundamental basis of RFP180’s anti-tumor effect could be attributed to the ubiquitin-dependent degradation of c-Myc (Ding et al., 2022).

#### EHD1

Downregulation of Eps15 homology domain 1 (*EHD1*) attenuated glycolysis and tumor growth in A549 cells, NCI-H1299 cells, and a xenograft mouse model. EHD1 promoted tumor progression through the activation of 14-3-3ζ/β-catenin/c-Myc signaling (Abuduwaili et al., 2022).

### Oncogenes *EGFR* and *KRAS* and their related molecules

Although EGFR and KRAS are associated with many tumors and have been shown to play important roles in NSCLC, they are also very important in glycolysis regulation.

#### EGFR

Epidermal growth factor receptor (*EGFR*) has been identified as an oncogene in recent decades. About 20% *EGFR* activation mutations have been identified in advanced NSCLC cases (Dogan et al., 2012). Autophagy caused by c-Jun N-terminal kinase (JNK) induces EGFR degradation, suggesting the therapeutic potential of the mitogen-activated protein kinase (MAPK)/JNK pathway in EGFR-activated NSCLC (Kim et al., 2018). The metabolic activity parameters MTV and TLG were used as predictors for TKI drug sensitivity and treatment outcomes in EGFR-mutant NSCLC patients (Jiang et al., 2022).

#### ALDOA

Aldolase A (ALDOA) presents high expression levels in NSCLC, particularly in squamous cell carcinoma. Fu et al. (2018) found that ALDOA promoted cell proliferation by increasing aerobic glycolysis through the activation of the EGFR/MAPK signaling pathway in H157 and H1299 cells.

#### CPS1

Pham-Danis et al. (2019) found that inhibition of the urea cycle enzyme CPS1 synergistically enhanced the suppressing effects of EGFR inhibitor on glycolysis and tumor growth in EGFR-mutant PC9 and HCC4006 cell lines.

#### PFKFB3

6-Phosphofructo-2-kinase/fructose-2,6-bisphosphatase 3 (PFKFB3), a regulator of EGFR signaling, is overexpressed in *EGFR*-mutant cells in PC9 and HCC827. The exposure of EGFR tyrosine kinase inhibitor (TKI) in H522 and PC9 cells led to the upregulation of PFKFB3 expression. The inhibition of both EGFR (TKI) and PFKFB3 (PFK158) enhanced anti-tumor efficacy in NSCLC (Lypova et al., 2019). Thus, the combination of PFKFB3 inhibitor and EGFR TKI is a prospective treatment strategy in NSCLC.

#### PDK1

Similarly, the pyruvate dehydrogenase kinase 1 (PDK1) inhibitor Cpd64, when combined with EGFR TKI, improved anti-cancer effects in *EGFR*-mutant NCI-H1975 and NCI-H1650 cell lines and in a xenograft mouse model *via* the improvement of oxidative phosphorylation and mitochondrial respiration (Wang F. et al., 2021).

#### KRAS

*KRAS* mutations have been discovered in multiple cancer types, including NSCLC, and are related to cancer metabolism reprogramming (e.g., promotion of glutaminolysis and glycolysis) (Kawada et al., 2017). *KRAS (G12D)* mutation significantly increased glycolysis-associated gene expression and resulted in enhanced glucose uptake and lactate generation in mouse embryonic fibroblasts (Kerr et al., 2016). Kim et al. reported that *KRAS* and tumor suppressor serine/threonine kinase 11 (*LKB1/STK11*) co-mutations had high risks of metastasis and metabolic rewiring through activation of the hexosamine biosynthesis pathway, according to data from metabolome and transcriptome profiles in mouse and human tumors. Furthermore, the silencing of *GFPT2* expression inhibited the cell growth and tumor survival in *KRAS/LKB1*-co-mutant H460 and H2122 cells and co-mutant mice (Kim et al., 2020). Emanuela Pupo et al. found that *KRAS* mutants presented distinctive metabolic profiles compared with wild types in tumors (Pupo et al., 2019). Therefore, glycolysis metabolism-related enzymes are potential therapeutic targets for *KRAS*-mutant NSCLC.

### Immune- and inflammation-related molecules

#### PD-1/PD-L1

PD-1/PD-L1 was detected to be overexpressed in NSCLC patients, and high expression of F-FDG PET/CT and PD-L1 was positively correlated with poor disease free survival (DFS) (Grizzi et al., 2019; Wang et al., 2020). In addition, high expression of PD-1/PD-L1 in cancer cells and tumor infiltrating lymphocytes (TILs) was negative-correlated with lactate dehydrogenase 5 (LDH5) and positive-correlated with HK2 and monocarboxylate transporter 2 (MCT2) Thus, PD-1/PDL-1 accompanied with glycolysis related markers, such as LDH5, act as prognostic and immunotherapy-outcome predictors in NSCLC (Giatromanolaki et al., 2019).

#### ISLR

High expression of leucine-rich repeat (ISLR) was associated with lower survival rates in NSCLC patients. The silencing of ISLR suppressed glycolysis, proliferation, invasion, and migration in A549 cells by activating the IL-6/JAK/STAT3 signaling pathway (Zhang P. et al., 2021).

#### NALP3

Inflammasome activator NLR family pyrin domain containing 3 (NALP3) depletion switched glucose metabolism from aerobic glycolysis to oxidative phosphorylation through interaction with DNA methyltransferase 1 associated protein 1 (DMAP1), which modulated transcription regulator DNA (cytosine-5)-methyltransferase 1 (DNMT1) in H1299 and A549 cells (He et al., 2020).

### Cell survival-related molecules

#### ENO1

The high expression of glycolytic enzyme alpha-enolase (ENO1), a key biomarker in tumor glycolytic metabolism, dramatically promoted cell growth and migration in A549 cells by activating the FAK/PI3K/AKT signaling pathway (Fu et al., 2015).

#### Barx2

The expression of Human BarH-like homeobox 2 (Barx2), a tumor suppressor linked to the Wnt/β-catenin signaling pathway, was relatively lower in tumor samples than in adjacent samples in NSCLC patients, and low Barx2 expression is associated with poor prognosis. Moreover, aerobic glycolysis was enhanced by the downregulation of Barx2, leading to increased cell proliferation, migration, and invasion in A549 cells (Chen et al., 2018).

#### TRIM59

The downregulation of the oncogene tripartite motif-containing 59 (TRIM59) reduced glycolysis and reversed cisplatin resistance in A549 cells *via* the suppression of the PTEN ubiquitination and the inhibition of AKT/HK2 activities (He and Liu, 2020).

#### Hsp27

Argpyrimidine is an advanced glycation end product resulting from the high glycolysis rate in tumor cells. Argpyrimidine-modified heat shock protein 27 (Hsp27) facilitated apoptosis evasion in SW1573 cells (van Heijst et al., 2006).

### Ubiquitin-related molecules

#### JOSD2

Krassikova et al. (2021) found that Josephin domain containing 2 (JOSD2) facilitated the proliferation of A549 cells by deubiquitinating the metabolic enzyme complex of ALDOA, phosphofructokinases PFK-1 and PFKL, and phosphoglycerate dehydrogenase (PHGDH).

#### OTUB2

Similarly, the deubiquitinating enzyme OTU deubiquitinase, ubiquitin aldehyde binding 2 (OTUB2) is a glycolytic stimulator which indicates poor survival outcome in NSCLC patients. Overexpression of OTUB2 enhanced glycolysis and promoted cell growth, migration, and invasion *via* the AKT/mTOR signaling pathway in A549 and H1299 cells and a xenograft mouse model (Li J. et al., 2019).

### Others

**FIGURE 2:**
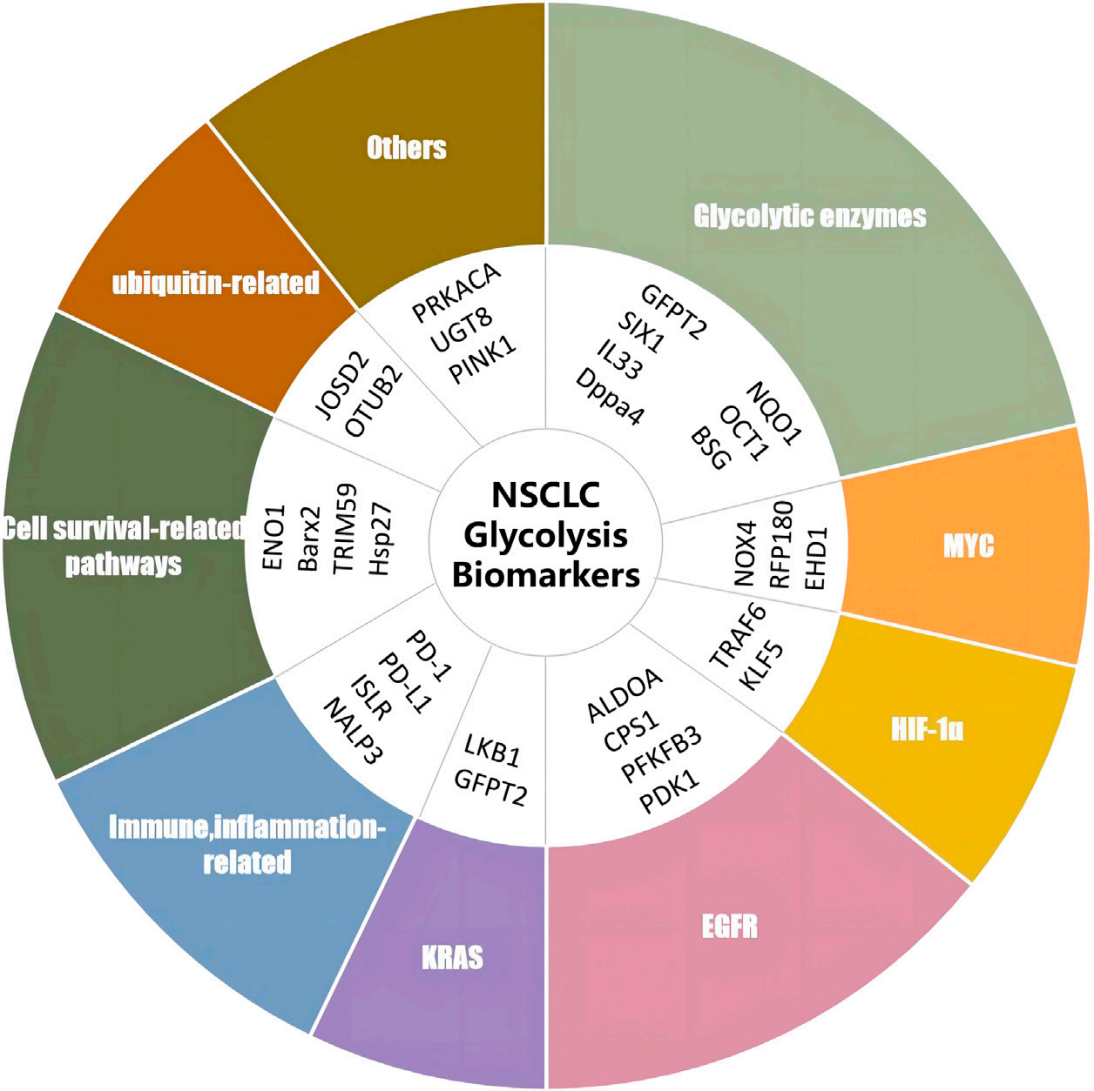
Biomarkers of glycolysis in NSCLC. RNF180, ring finger protein 180; UGT8, UDP-glycosyltransferase 8; SIX1, sine oculis homeobox homolog 1; TRAF6, TNF receptor-associated factor 6; EHD1, Eps15 homology domain 1; OCT-1, octamer-transcription factor-1; ISLR, leucine-rich repeat; JOSD2, Josephin domain containing 2; PRKACA, protein kinase cAMP-activated catalytic subunit alpha; PFKP, phosphofructokinase; TRIM59, tripartite motif-containing 59; NALP3, NLR family pyrin domain containing 3; CPS1, carbamoyl phosphate synthetase I; PFKFB3, 6-phosphofructo-2-kinase/fructose-2,6-bisphosphatase; PD-1, programmed cell death protein 1; PD-l1, programmed death-ligand 1; OTUB2, OTU deubiquitinase, ubiquitin aldehyde binding 2; DPPA4, developmental pluripotency-associated 4; PINK1, PTEN-induced putative kinase 1; KLF5, factor 5; ALDOA, aldolase A; EGFR, epidermal growth factor receptor; GFPT2, glutamine-fructose-6-phosphate transaminase 2; NQO1, NAD(P)H quinone oxidoreductase 1; PDK1, pyruvate dehydrogenase kinase 1; BARX2, BarH-like homeobox 2; PGC-1alpha, pPARG coactivator 1 alpha; NOX4, NADPH oxidase 4; IL-33, interleukin 33; ENO1, alpha-enolase; BSG, cD147/basigin; KRAS, Kirsten-RAS; GAPDH, glyceraldehyde-3-phosphate dehydrogenase; FBP1, fructose-1,6-bisphosphatase; HIF-1α, hypoxia-inducible factor 1 subunit alpha; Hsp27, heat shock protein 27.

Apart from the diverse biomarkers described earlier, a variety of additional targets and underlying mechanisms have been reported to be correlated with glycolysis in NSCLC. Protein kinase cAMP-activated catalytic subunit alpha (PRKACA) enhances glycolysis by activating triosephosphate isomerase (TPI) serine 58 (Ser58) (Duan et al., 2021). Ji et al. (2022) found that UDP-glycosyltransferase 8 (UGT8) enhanced tumor growth by promoting glycolysis in A549 cells, H460 cells, and a xenograft mouse model, and the depletion of UGT8 diminished tumor malignancy *in vitro* and *in vivo*. The depletion of PTEN-induced putative kinase 1 (PINK1) *via* shRNA in A549 cells suppressed tumor malignancy and increased the sensitivity of glycolysis inhibitor 3-BP by disrupting ATP production, promoting ROS generation, and inducing apoptosis (Dai et al., 2019). The glycolysis-related biomarkers reported earlier are summarized in Table 1 and Figure 2.

**TABLE 1:** Glycolysis-related biomarkers in NSCLC.

No.	Target	Related target and pathway	Publication year	DOI
1	RING finger protein 180 (RNF180)	c-Myc	2022	10.1186/s12957-022-02599-x
2	UDP-glycosyltransferase 8(UGT8)	Transcription factor SOX9	2022	10.1016/j.bbrc. 2021.11.099
3	Sine oculis homeobox homolog 1 (SIX1)	HK2/LDHA	2022	10.3892/ol. 2022.13304
4	TNF receptor-associated factor 6 (TRAF6)	Akt-HIFα Pathway	2021	10.1155/2021/3431245
5	Eps15 homology domain 1 (EHD1)	β-catenin/c-Myc signaling	2021	10.1016/j.canlet. 2021.06.023
6	Octamer-transcription factor-1 (OCT-1)	HK2	2021	10.1007/s11010-021-04171-9
7	Leucine-rich repeat (ISLR)	IL-6/JAK/STAT3 signaling pathway	2021	10.3892/ijmm. 2021.5055
8	Josephin domain containing 2 (JOSD2)	Deubiquitinase	2021	10.1038/s41418-020-00639-1
9	Protein kinase cAMP-activated catalytic subunit alpha(PRKACA)	TPI Ser58	2021	10.1016/j.celrep. 2021.110137
10	Phosphofructokinase (PFKP)	Rate-limiting enzyme	2021	10.3892/mmr. 2020.11712
11	Tripartite motif-containing 59 (TRIM59)	PTEN/AKT/HK2	2020	10.1016/j.gene. 2020.144553
12	NLR Family Pyrin Domain Containing 3 (NALP3)	DNMT1	2020	10.1016/j.lfs. 2019.117165
13	Carbamoyl phosphate synthetase I (CPS1)	Urea cycle enzyme	2019	10.1158/1541-7786.Mcr-18-1068
14	6-Phosphofructo-2-kinase/fructose-2,6-bisphosphatase (PFKFB3)	EGFR signaling	2019	10.1074/jbc.RA119.007784
15	Programmed cell death protein 1 (PD-1)/PD-L1	Immune checkpoint	2019	10.1007/s12032-019-1299-4
16	OTU deubiquitinase, ubiquitin aldehyde binding 2 (OTUB2)	Deubiquitinating enzymes	2019	10.7150/thno.29545
17	Developmental pluripotency-associated 4 (DPPA4)	LDHB	2019	10.3892/mmr. 2019.10272
18	PTEN-induced putative kinase 1 (PINK1)	ROS	2019	10.1016/j.pharep. 2019.08.002
19	Krueppel-like factor 5 (KLF5)	HIF-1α and PI3K/Akt/mTOR pathway	2018	10.1186/s12967-018-1543-2
20	Aldolase A (ALDOA)	EGFR/MAPK pathway	2018	10.1186/s40880-018-0290-3
21	Epidermal growth factor receptor (EGFR)	EGFR	2018	10.1158/0008-5472.Can-18-0117
22	Glutamine-fructose-6-phosphate transaminase 2 (GFPT2)	Rate-limiting enzyme of the hexosamine biosynthesis pathway (HBP)	2018	10.1158/0008-5472.Can-17-2928
23	NAD(P)H:quinone oxidoreductase 1 (NQO1)	HK2	2018	10.1016/j.bbrc. 2017.12.160
24	Pyruvate dehydrogenase kinase 1 (PDK1)	EGFR	2018	10.1016/j.ejphar. 2018.09.016
25	BarH-like homeobox 2 (Barx2)	Wnt/β-catenin pathway	2018	10.1111/1759-7714.12593
26	PPARG coactivator 1 alpha (PGC-1alpha)	GAPDH	2017	10.1038/s41598-017-17009-6
27	NADPH oxidase 4 (NOX4)	c-Myc and ROS/PI3K/Akt signaling pathway	2016	10.1016/j.freeradbiomed. 2016.10.500
28	Interleukin 33 (IL-33)	GLUT1/IL-33/ST2 pathway	2016	10.1016/j.bbrc. 2016.09.081
29	Alpha-enolase (ENO1)	FAK-mediated PI3K/AKT pathway	2015	10.1186/s13045-015-0117-5
30	CD147/BASIGIN (BSG)	MCT1/4	2015	10.18632/oncotarget.2862
31	Kirsten-Ras (KRAS)	(GAPDH/PKM2/LDH-A/LDH-B) and pentose phosphate pathway (PPP)	2014	10.1021/pr500327v
32	Glyceraldehyde-3-phosphate dehydrogenase (GAPDH)	GAPDH	2013	10.1097/CAD.0b013e32835e3378
33	Fructose-1,6-bisphosphatase (FBP1)	GAPDH-associated cell cycle (GACC)	2013	10.1371/journal.pone.0061262
34	Hypoxia-inducible factor 1 subunit alpha (HIF-1α)	Glycolysis pathway	2006	10.1186/1471-2407-6-26
35	Heat shock protein 27 (Hsp27)	Apoptosis	2006	10.1016/j.canlet. 2005.10.042

## Current treatments and candidates targeting glycolysis in NSCLC

**FIGURE 3:**
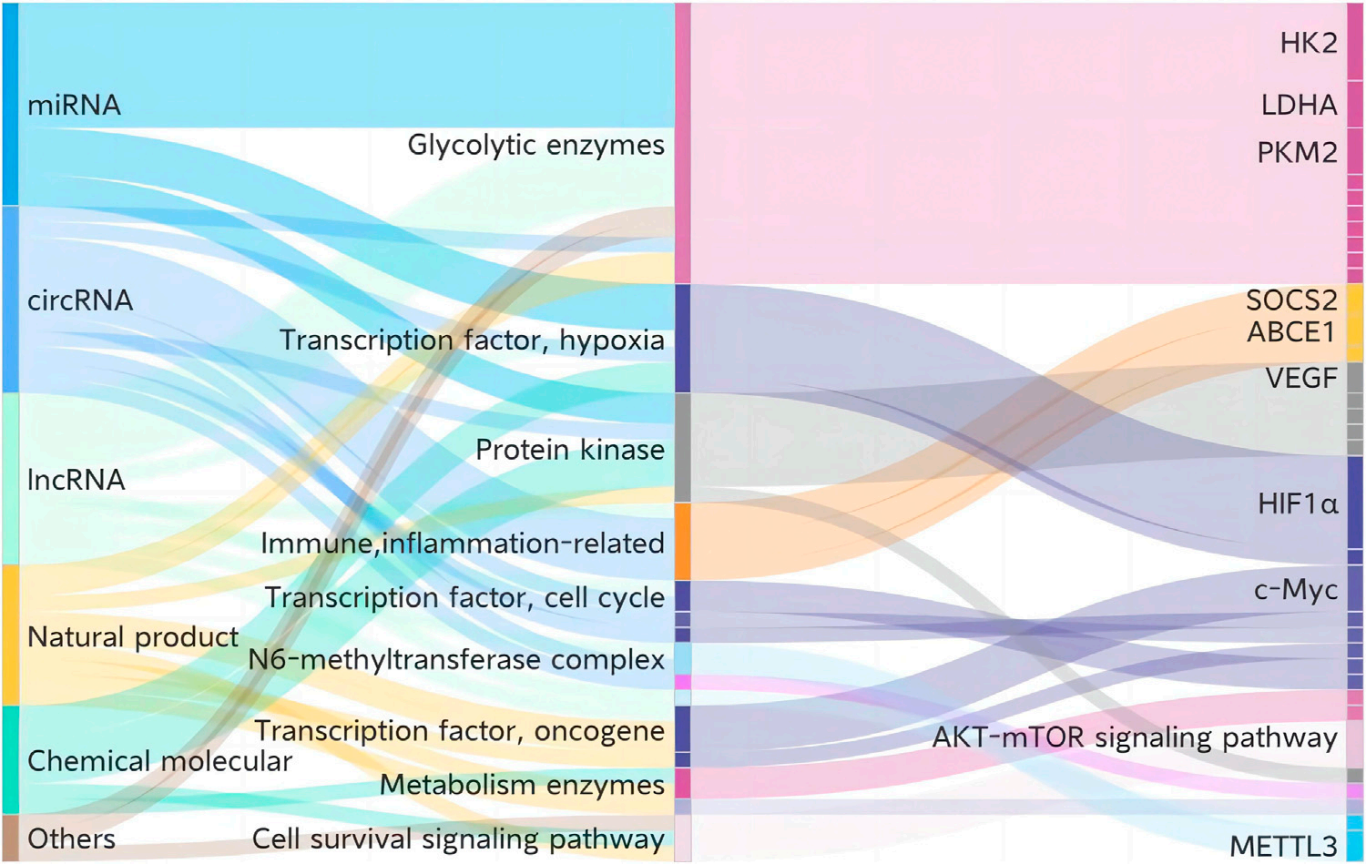
Current treatments and candidates targeting glycolysis in NSCLC. HK2, hexokinase 2; PKM2, pyruvate kinase M2; LDHA, lactate dehydrogenase A; SOCS2, suppressor of cytokine signaling 2; ABCE1, ATP-binding cassette subfamily E member 1; VEGF, vascular endothelial growth factor; HIF-1α, hypoxia-inducible factor 1 subunit alpha; c-Myc, MYC proto-oncogene; METTL3, methyltransferase 3.

Despite the insufficiencies of metabolic treatment in NSCLC, various current treatments and candidates—including chemotherapy, targeting therapy, anti-tumor small molecules, biopharmaceutical therapy, and natural products—present potent interventions in glycolysis. We summarize relevant details drawn from previous studies in Tables 2‐4 and Figure 3.

**TABLE 2:** Small molecules and novel preparations targeting glycolysis in NSCLC.

No.	Drug name	Target	Publication year	Category	DOI
1	Caudatin	Raf/MEK/ERK pathway	2022	Chemical molecular	10.1080/13880209.2022.2050768
2	Crizotinib	Anaplastic lymphoma kinase (ALK) inhibitor	2021	Chemical molecular	10.1111/1759-7714.14184
3	Gefitinib	EGFR	2020	Chemical molecular	10.12122/j.issn.1673-4254.2020.06.17
4	Dichloroacetate (DCA)	HIF-3α	2020	Chemical molecular	10.1155/2020/3176375
5	Fenbendazole	Acetylcholinesterase (AChE)	2018	Chemical molecular	10.1038/s41598-018-30158-6
6	Albendazole	HIF-1α/VEGF	2017	Chemical molecular	10.1007/s11010-016-2927-3
7	Gal-Pt	GLUTs	2016	Chemical molecular conjugate	10.1016/j.ejmech. 2016.01.016
8	Ad-apoptin	AMPK/mTOR signaling pathway	2021	Oncolytic adenovirus	10.1016/j.yexcr. 2021.112926
9	siRNA and DTX	Phosphoglycerate mutase 1 (PGAM1)	2021	Nanovesicle	10.1186/s12951-021-01085-y

**TABLE 3:** Non-coding RNAs targeting glycolysis in NSCLC.

No.	RNA name	Target	Publication year	Category	DOI
1	miR-16-5p	LDHA	2022	miRNA	10.1016/j.lfs. 2022.120722
2	miR-206	HK2	2020	miRNA	10.1093/jb/mvz099
3	miR-449a	LDHA	2018	miRNA	10.3727/096504017 × 15016337254605
4	miR-182	HIF1α	2018	miRNA	10.1016/j.bbrc. 2018.06.035
5	miR-214	HK2/PKM2	2018	miRNA	10.1016/j.biopha. 2018.06.009
6	miR-124	AKT1/GLUT1/HK2	2018	miRNA	10.1177/1010428317706215
7	miR-512-5p	Cyclin-dependent kinase inhibitor p21	2016	miRNA	10.3892/ijo. 2015.3279
8	miR-21	HIF1α	2016	miRNA	10.3892/mmr. 2016.5010
9	miR-199a	HIF1α	2013	miRNA	10.1007/s11010-013-1795-3
10	miR-143	HK2	2012	miRNA	10.1074/jbc.M112.373084
11	circ_0008797	miR-301a-3p/SOCS2	2022	circRNA	10.1002/tox.23518
12	circ_0016760	miR-4295/E2F3	2022	circRNA	10.1089/cbr. 2020.3621
13	circ_0020123	miR-193a-3p/IRF4	2022	circRNA	10.4149/neo_2022_211013N1449
14	circEHD2	miR-3186-3p/FOXK1	2022	circRNA	10.1080/21655979.2022.2031385
15	circ_0006677	miR-578/SOCS2	2021	circRNA	10.3389/fphar. 2021.657053
16	circ_0000517	miR-330-5p/YY1	2021	circRNA	10.1002/kjm2.12440
17	circPUM1	miR-590-5p/METTL3	2021	circRNA	10.1080/15384101.2021.1934625
18	circ_0000735	miR-635/FAM83F	2021	circRNA	10.1080/01902148.2021.1881188
19	circSLC25A16	miR-488-3p/HIF-1α/LDHA	2020	circRNA	10.1038/s41419-020-2635-5
20	circ-ACACA	miR-1183/BCL-2	2020	circRNA	10.3892/ijmm. 2020.4549
21	circ_0002130	miR-498/ABCE1	2020	circRNA	10.2147/ott.S243214
22	lnc-CYB561-5	HK2/PFK1	2022	lncRNA	10.1111/jcmm.17057
23	ABHD11-AS1	METTL3	2021	lncRNA	10.1002/jcp.30023
24	HOTAIRM1	miR-498/ABCE1	2021	lncRNA	10.1007/s13258-021-01052-9
25	LINC00243	miR-507/PDK4	2020	lncRNA	10.1007/s11010-019-03635-3
26	AC020978	PKM2/HIF1α	2020	lncRNA	10.7150/thno.43839
27	BCYRN1	miR-149/PKM2	2020	lncRNA	10.3892/mmr. 2020.10944
28	HOTTIP	miR-615-3p/HMGB3	2019	lncRNA	10.1016/j.ejphar. 2019.172615
29	NORAD	miR-136-5/E2F1	2019	lncRNA	10.3892/mmr. 2019.10210
30	LINC01123	miR-199a-5p/c-Myc	2019	lncRNA	10.1186/s13045-019-0773-y

**TABLE 4:** Natural products targeting glycolysis in NSCLC.

No.	Drug name	Target	Publication year	Category	DOI
1	Shikonin	PFKFB/PKM2 pathway	2022	Natural compound	10.1080/21655979.2022.2086378
2	Dihydroartemisinin	ERK/c-Myc pathway	2022	Natural compound	10.1016/j.bcp. 2022.114941
3	Tanshinone IIA	Sine oculis homeobox homolog 1 (SIX1)	2022	Natural compound	10.3892/ol. 2022.13304
4	Ligustilide	PTEN/AKT signaling pathways	2021	Natural compound	10.1016/j.taap. 2020.115336
5	α-Hederin	SIRT6	2021	Natural compound	10.1080/13880209.2020.1862250
6	β-Elemene	Adenosine monophosphate-activated protein kinase α (AMPKα)	2020	Natural compound	10.1042/bsr20194389
7	Piperlongumine	HK2	2019	Natural compound	10.7150/ijbs.31749
8	Jolkinolide B	HK2/Akt/mTOR pathway	2018	Natural compound	10.1002/jcb.26742
9	Triptolide	HK2/Akt/mTOR pathway	2018	Natural compound	10.1016/j.biopha. 2018.04.198
10	Deguelin	HK2/AKT1	2017	Natural compound	10.18632/oncotarget.15937
11	Resveratrol	HK2/AKT1	2016	Natural compound	10.1016/j.yexcr. 2016.11.002
12	Oroxylin A	HK2/c-Src	2013	Natural compound	10.1016/j.bbagen. 2013.03.009
13	Shenmai injection (SMI)	AKT-mTOR-c-Myc signaling pathway	2020	Natural extract	10.1155/2020/9243681
14	Water-extract branch from Cinnamomum cassia Blume	Pyruvate dehydrogenase kinase (PDHK)	2018	Natural extract	10.1016/j.jphs. 2018.10.005
15	Hydroalcoholic extract from the leaves of *Nerium oleander*	Suppression of glycolysis	2013	Natural extract	10.1055/s-0032-1328715

### Chemotherapy and its resistance

Chemotherapy is the mainstream treatment approach in most cancer types due to its significant effectiveness. Glycolysis is revealed to be correlated with the clinical outcomes of chemotherapy. According to gene polymorphism research in NSCLC patients, the glycolysis-related genes phosphofructokinase liver type (PFKL) and glucose phosphate isomerase (GPI) are correlated with outcomes of patients treated with first-line paclitaxel–cisplatin therapy, and genetic polymorphism in ATP-binding cassette subfamily B member 1 (ABCB1) and HIF-1α predicted the efficacy of paclitaxel-carboplatin therapy (Park et al., 2016; Choi et al., 2020). Galactose-conjugate (trans-R,R-cyclohexane-1,2-diamine)-2-chloromalonato-platinum (II) complex (Gal-Pt) outperformed oxaliplatin and cisplatin in antitumor activity by affecting the glucose uptake target GLUTs in H460 cells and a xenograft model (Wu et al., 2016). These studies indicate that glycolysis-related genes could be adopted as prediction factors for chemotherapy outcomes and as potential targets for the enhancement of drug effectiveness.

Moreover, glycolysis is associated with chemotherapy resistance. The molecule 5-Fu, a pyrimidine analog for pan-cancer treatment, activated glycose metabolism in resistant A549 cells, while cisplatin-resistant cells suppressed glycose metabolism (Zhao et al., 2014). Hypoxia-induced cisplatin-resistant A549 cells featured a high expression of PKM2, which contributed to tumor glycose metabolism reprogramming and promoted acidic TME and cell proliferation in NSCLC cells A549, accelerating cisplatin resistance effects (Wang D. et al., 2021).

### Targeting therapy

Various small molecules or antibodies which specifically target the biomarkers of glycolysis have been investigated as NSCLC-targeting treatments. Gefitinib, a tyrosine kinase inhibitor (TKI), suppressed NSCLC cell growth by impairing cellular glycolysis and inhibiting the PI3K-Akt-mTOR signaling pathway in A549 and H1975 cells (Zhou et al., 2020). The efficacy of the anaplastic lymphoma kinase (ALK) inhibitor crizotinib was suspended by HK2-mediated glycolysis, and the inhibition of glycolysis and AKT/mTOR signaling pathway dramatically improved crizotinib sensitivity in both ALK (+) H3122 and H2228 cells (Lin et al., 2021). As an oncogene, AKT kinase was overexpressed in multiple tumors, including NSCLC, and facilitated glycolysis metabolism in tumor cells (Elstrom et al., 2004). The combination of AKT inhibitor and microtubule-targeting agents (MTAs) caused a synergic anti-tumor effect in multiple paclitaxel-resistant or anti-EGFR-resistant NSCLC cell lines (A549, A549/EpoB40, HCC827, H1650, and H1975 cells) and human NSCLC xenografts (Le Grand et al., 2017). The combination of glycolysis inhibitor 2-deoxy-d-glucose (2DG) with the EGFR TKI afatinib enhanced the sensitivity of H1975 and PC9-GR cells with an EGFR T790M mutation by inhibiting glycolysis through suppression of the AMPK/mTOR/Mcl-1 signaling pathway (Kim et al., 2013). The molecule 5-(n-(8-methoxy-4-quinolyl) amino) pentyl nitrate (5MPN), a selective inhibitor of glycolytic regulatory enzyme 6-phosphofructo-2-kinase/fructose-2,6-bisphosphatase 4 (PFKFB4), effectively attenuated glycolysis and tumor growth in C57BL mice, with high oral bioavailability (Chesney et al., 2015). THZ1, a cyclin-dependent kinase 7 (CDK7) inhibitor, induced cell cycle arrest and apoptosis in H1299, A549, H292, and H23 NSCLC cells, which is attributed to the blockage of glycolysis-related pathways (Cheng et al., 2019). These studies indicate the promising therapeutic efficacy of cancer metabolism intervention, along with targeting therapy.

### Anti-tumor small molecules

Methyl N-(6-phenylsulfanyl-1H-benzimidazol-2-yl) carbamate (fenbendazole, FZ), a microtubule-interfering agent, significantly inhibited the glycose uptake genes *GLUT* and *HK2* in A549 cells and impeded the growth of human xenografts in a nu/nu mice model (Dogra et al., 2018). Albendazole (ABZ), a broad-spectrum benzimidazole carbamate anthelminthic and a microtubule inhibitor, presented similar anti-glycolysis effects with FZ by intervening in HIF-1α-dependent glycolysis. ABZ suppressed A549 and H1299 NSCLC cell growth and decreased VEGF and HIF-1α expression in an A549 xenograft mouse model (Zhou et al., 2017). These studies confirm the critical role of glycolysis intervention and tumor-microenvironment metabolism in NSCLC treatment.

The metabolic agent dichloroacetate (DCA), an inhibitor of pyruvate dehydrogenase, has demonstrated therapeutic potential in NSCLC. Aerobic glycolysis was ameliorated in DCA-treated A549 and H1299 cells, with decreased lactate generation and glucose consumption (Allen et al., 2015). The combination of angiogenesis inhibitor 2-methoxyestradiol (2-ME) with DCA showed synergistic anti-tumor effects in A549 cells by reducing the signaling transduction of HIF-3α and, partially, of HIF-1α (Romero et al., 2020). The combination of the energy metabolism inhibitor 3-bromopyruvate (3-BrPA) with the mTOR inhibitor rapamycin synergistically suppressed cell growth in H1299 and H23 cell lines and tumor growth in an A/J mouse model, respectively, indicating the promising therapeutic potential of both mTOR signaling and glycolysis inhibition in NSCLC (Zhang et al., 2015). Therefore, multiple anti-tumor small molecules increase their effects in NSCLC by directly or indirectly targeting the regulation of tumor glycolysis metabolism. Intervention in glycolysis could not only induce cell apoptosis but also reverse drug resistance. The anti-tumor small molecules discussed earlier are listed in Table 2.

### Biopharmaceutical therapy

The development of biotechnologies and biopharmaceutics for cancer treatment has grown rapidly in recent years. Ad-apoptin is an oncolytic adenovirus that impairs AMPK-dependent glycolysis in NSCLC. Ad-apoptin inhibited tumor cell invasion and migration in A549 and NCI-H23 cells, as well as suppressing tumor growth in nude mice. The mechanism underlying the anti-NSCLC effect of ad-apoptin is the inhibition of glucose uptake and lactate generation through suppression of the AMPK/mTOR signaling pathway (Song et al., 2021).

Zhang et al. developed an effective TME biomimetic nanoplatform for NSCLC treatment, which was a hybrid nanovesicle loaded with siRNA against glycolytic regulator phosphoglycerate mutase 1 (PGAM1) and the chemotherapeutic drug docetaxel (DTX). This pH-driven siRNA and DTX hybrid nanoplatform ensured precise drug release and synergistic glycolysis impairment in A549 cells and impeded tumor growth in A549-cell tumor-bearing nude BALB/c mice (Zhang W. et al., 2021). Moreover, non-coding RNAs including microRNA (miRNA), circular RNA (circRNA), and long non-coding RNA (lncRNA) have been widely studied for the impairment of glycolysis in NSCLC. Most miRNAs have targeted key glycolytic enzymes such as HK2 (miR-143 and miR-206) (Fang et al., 2012; Jia et al., 2020), LDHA (miR-449a and miR-16-5p) (Li et al., 2018b; Arora et al., 2022), and HIF1α (miR-21, miR-182, and miR-199a) (Ding et al., 2013; Jiang et al., 2016; Wang et al., 2018). The circRNA and lncRNA target sequences have presented diverse glycolytic biomarkers under various molecular mechanisms in NSCLC. For instance, the activation of circ_0008797 showed anti-glycolysis effects in A549 and H1229 cells, as well as in a nude mouse model, by sponging miR-301a-3p and targeting suppressor of cytokine signaling 2 (SOCS2) (Abuduwaili et al., 2022). The lncRNA HOXA transcript antisense RNA myeloid-specific 1 (HOTAIRM1), which sponges the miR-498/ATP-binding cassette subfamily E member 1 (ABCE1), attenuated glycolysis metabolism in NCI-H1299 and A549 cells (Chen et al., 2021). Additionally, the regulation of miR-498 might reverse the resistance effects of osimertinib through the inhibition of the key glycolytic enzymes GLUT1, HK2, and LDHA in NCI-H1299 and A549 cells (Ma et al., 2020). Thus, various biologics have been reported to be correlated with the regulation of intracellular glycolytic metabolism. Although most of the previous research studies have been conducted *in vitro*, biopharmaceutics targeting glycolysis are a promising strategy for cancer treatment. The information detailed earlier is listed in Table 3.

### Natural products

Natural compounds or extracts from animals, plants, or minerals, have been shown to have multiple medicinal and pharmacological activities. Natural products are a precious library for anti-tumor drug discovery, which has been a cutting-edge research field in the recent decades. In research into NSCLC drug candidate discovery, various natural products have been identified with potential for NSCLC treatment *via* their anti-glycolysis activities. Shikonin, an active ingredient derived from *Lithospermum erythrorhizon*, dampened intracellular glycolysis in A549 and PC9 cells. Shikonin improved the sensitivity of cisplatin in NSCLC mice by inhibiting the key glycolytic enzyme PKM2. The combination of shikonin with cisplatin synergistically inhibited PKM2 and GLUT1, according to data from immune-histological assays of tumor tissues. Furthermore, shikonin affected the glycolysis regulator PFKFB2 at the transcriptome level in A549 and H446 cells (Sha et al., 2021; Dai et al., 2022). Alpha-hederin is derived from the *Pulsatilla chinensis* (Bunge) Regel (Ranunculaceae). Fang et al. (2021) found *a*-hederin to demonstrate anti-proliferation effects in A549, NCI-H460, and NCI-H292 cells and in a xenograft mouse model by suppressing sirtuin 6 (SIRT6)-correlated glycolysis. The natural flavonoid deguelin is extracted from *Derris trifoliata* Lour, while resveratrol is an active ingredient derived from *Veratrum grandiflorum*. Deguelin and resveratrol have been studied in multiple cancer types for years and are well-known anti-tumor compounds with inhibitory properties against AKT1 and HK2-mediated glycolysis in the human NSCLC cell lines H460, H1650, and HCC827 (Li et al., 2016; Li et al., 2017). Jolkinolide B (JB), triptolide, and oroxylin A are bioactive compounds extracted from *Euphorbia fischeriana* Steud, *Tripterygium wilfordii* Hook F, and *Scutellariae radix,* respectively. These three natural compounds exhibited anti-metastasis effects in H1299, NCI- H460, and A549 cells and in nude mice, and the underlying mechanism is their intervention in intracellular glycolysis and the AKT/mTOR pathway (Wei et al., 2013; Gao and Han, 2018; Hamdi et al., 2018). Ligustilide, a derivative of *Angelica sinensis*, inhibited cell proliferation in H1299 and A549 and attenuated tumor growth in nude mice *via* inhibition of the PTEN/AKT signaling pathway (Jiang et al., 2021). The antimalarial drug dihydroartemisinin (DHA), which is extracted from *Artemisia annua*, showed cytotoxicity in diverse tumor models, including NSCLC. DHA inhibited glucose uptake and glycolysis in NCI-H358, A549, and PC-9 cells by downregulating GLUT1, mTOR, and S6 ribosomal protein through suppression of the ERK/c-Myc signaling pathway (Mi et al., 2015; Zhang et al., 2022). Beta-elemene, which is an essential oil of *Curcuma wenyujin*, inhibited miR-301a-3p-induced Warburg effects in NSCLC cell line NCI-H1650 by activating adenosine monophosphate-activated protein kinase *a* (AMPKα) (Li et al., 2020). Tanshinone IIA (Tan IIA), the active ingredient of *Salvia miltiorrhiza*, exhibited antitumor effects in NSCLC cells A549 and H292 and xenograft BALB/c-nu/nu nude mice by targeting the glycolytic mediator sine oculis homeobox homolog 1 (SIX1), which is a poor-prognosis predictor in NSCLC (Qi et al., 2022). The natural compound piperlongumine, an active component of *Piper longum L*, suppressed cell proliferation and colony formation in NSCLC cells HCC827 and H1975 by inhibiting cellular glycolysis and activating the apoptosis pathway. Additionally, the blockage of HK2 and AKT1 was essential for the anti-tumor effects of piperlongumine in a xenograft mouse model (Zhou et al., 2019).

Natural extracts have also presented effective anti-cancer effects in NSCLC through inhibition of glycolytic metabolism. Calderón-Montaño et al. (2013) found that hydroalcoholic extract from the leaves of *Nerium oleander*, a plant toxic to livestock, demonstrated anti-tumor effects in A549 cells through suppression of glycolysis. Lee et al. (2018) detected that water-extracted branch from *Cinnamomum cassia Blume*, which is widely used as a food additive or spice, suppressed aerobic glycolysis in A549 and H1299 cells *via* inhibiting pyruvate dehydrogenase kinase (PDHK), resulting in increased ROS and mitochondrial damage and consequently inducing cell apoptosis. Shenmai injection (SMI), which was developed from the medicinal Chinese herbs *Radix Ginseng Rubra* and *Radix Ophiopogonis*, is a commercially available drug for treating cardiovascular diseases. Sun et al. found that the combination of SMI with cisplatin increased the cisplatin sensitivity of resistant A549/DDP cells by suppressing glycolysis through inhibition of the AKT-mTOR-c-Myc signaling pathway (Sun Y. et al., 2020). Thus, natural products have shown inhibitory effects on glycolysis metabolism in cancer for multiple targets. However, the anti-tumor effects of most natural products against NSCLC have been determined *in vitro*. The combination of natural compounds with current therapies is a promising direction of studies, as well as *in vivo* study, and clinical study is needed in the future. Detailed information is given in Table 4.

## Conclusion and future perspectives

NSCLC is the leading cause of death in cancer. Glycolytic metabolism heterogeneity is a critical feature of tumor metabolism that can distinguish cancer cells from normal cells. The reprogramming of glucose metabolism is common in multiple tumors, including NSCLC. As in other tumors, glycolysis represents the main metabolic process in NSCLC, instead of oxidative phosphorylation. Thus, glycolysis is vital for tumor cell survival, and targeting the inhibition of glycolysis is promising in the treatment of NSCLC.

However, most of the key glycolytic enzymes are involved in the progression of NSCLC, which means that the scope of target selection is broad and its specificity difficult to guarantee. The identification of appropriate targets and biomarkers in glycolysis that are specific for NSCLC treatment is still a challenge at present. However, high expression of PDK1 is an independent prognostic factor in NSCLC. LDHB is a potential specific target since it has been reported as a positive survival predictor in NSCLC, although the exact process of the lactate metabolism is complicated and LDHB has also been revealed to be a risk factor in other tumor cell types. Targeting both MCT2 and GLUT1 might be promising in the treatment of the adenocarcinoma subtype of NSCLC. PFKM might also be a prognostic predictor in postoperative NSCLC patients. DPPA4, NQO1, GAPDH/MT-CO1, PGC-1α, OTUB2, ISLR, Barx2, OTUB2, and RFP180 might be biomarkers or prognostic predictors of NSCLC. In addition, natural compounds or extracts may be promising therapeutic approaches in targeting the multiple steps of glycolysis metabolism since natural products always present multi-target properties.

Although current therapies including chemotherapy, targeting therapy, and biopharmaceutical therapy have shown dramatic effectiveness in the treatment of NSCLC, partially through glycolysis suppression, the occurrence of drug resistance during treatment is a crucial concern. Targeting the enhancement of tumor glycolytic metabolism inhibition may also be beneficial in reversing drug resistance and enhancing the anti-tumor effects of current treatments in NSCLC. Thus, the combination of natural products, small molecules, and biopharmaceutics systems targeting the inhibition of glycolysis with current therapeutic approaches might be a promising strategy as *in vivo* and clinical studies accumulate in the future.

In conclusion, targeting glycolysis might be a potential therapeutic approach in NSCLC treatment, although finding specific targets and biomarkers is still challenging at present. The combination of glycolysis intervention with current therapeutic approaches might increase treatment efficacy in NSCLC patients.

## Glossary

ABCB1: ATP-binding cassette subfamily B member 1
ABCE1: ATP-binding cassette subfamily E member 1
ABZ: albendazole
AChE: acetylcholinesterase
ADP: adenosine diphosphate
ALDOA: aldolase A
ALK: anaplastic lymphoma kinase
AMPK: adenosine monophosphate-activated protein kinase
AMPKα: adenosine monophosphate-activated protein kinase *a*
ATP: adenosine triphosphate
Barx2: BarH-like homeobox 2
BSG: basigin
CDK7: cyclin-dependent kinase 7
circRNA: circular RNA
CPS1: carbamoyl phosphate synthetase I
CTC: circulating tumor cell
DCA: dichloroacetate
DHA: dihydroartemisinin
DPPA4: developmental pluripotency-associated 4
DTX: docetaxel
EGFR: epidermal growth factor receptor
EHD1: eps15 homology domain 1
ENO1: alpha-enolase
ETC: electron transfer chain
FBP1: fructose-1,6-bisphosphatase
FDG-PET: F-fluorodeoxyglucose positron emission tomography
5MPN: 5-(n-(8-methoxy-4-quinolyl) amino) pentyl nitrate
Gal-Pt: galactose-conjugate (trans-R,R-cyclohexane-1,2-diamine)-2- chloromalonato-platinum (II) complex
GAPDH: glyceraldehyde-3-phosphate dehydrogenase
GFPT2: glutamine-fructose-6-phosphate transaminase 2
GLUT1: glucose transporter 1
GPI: glucose phosphate isomerase
HIF-1α: hypoxia-inducible transcription factor-1 alpha
HK: hexokinase
Hsp27: heat shock protein 27
IL-33: interleukin 33
ISLR: leucine-rich repeat
JB: jolkinolide B
JNK: c-Jun N-terminal kinase
JOSD2: Josephin domain containing 2
KLF5: Krũppel‐like factor 5
KRAS: Kirsten RAS
LDH: lactate dehydrogenase
lncRNA: long non-coding RNA
MAPK: mitogen-activated protein kinase
MCT1-4: monocarboxylate transporter 1-4
miRNA: microRNA
MTAs: microtubule-targeting agents
MTH1: MutT homolog 1
mTOR: mammalian target of rapamycin
MTV: metabolic tumor volume
NADH: nicotinamide adenine dinucleotide
NALP3: NLR family pyrin domain containing 3
NOX4: NADPH oxidase 4
NQO1: NADPH quinone oxidoreductase 1
NSCLC: non-small cell lung cancer
OCT1: octamer-transcription factor 1
OTUB2: OTU deubiquitinase, ubiquitin aldehyde binding 2
PD-1: programmed cell death protein 1
PDK1: pyruvate dehydrogenase kinase 1
PFKFB3: 6-phosphofructo-2-kinase/fructose-2,6-bisphosphatase 3
PFKL: phosphofructokinase, liver type
PFKM: phosphofructokinase, muscle type
PFKP: phosphofructokinase
RFP180: ring finger protein 180
PGAM1: phosphoglycerate mutase 1
PINK1: PTEN-induced putative kinase 1
PI3K: phosphoinositide 3-kinase
PKLR: liver and red blood cell isoform
PKM1: muscle isoform pyruvate kinase M1
PKM2: pyruvate kinase M2
PRKACA: protein kinase cAMP-activated catalytic subunit alpha
ROS: reactive oxygen species
SCLC: small cell lung cancer
SIX1: sine oculis homeobox homolog 1
SOCS2: suppressor of cytokine signaling 2
STK11: serine/threonine kinase 11
Tan IIA: tanshinone IIA
TCA: tricarboxylic acid
TKI: tyrosine kinase inhibitor
TLG: total lesion glycolysis
TME: tumor microenvironment
TRAF6: TNF receptor-associated factor 6
TRIM59: tripartite motif-containing 59
2DG: 2-deoxy-d-glucose
2-ME: 2-methoxyestradiol
3-BrPA: 3-bromopyruvate
UGT8: UDP-glycosyltransferase 8
VEGF: vascular endothelial cell growth factor
